# Blockade of IL-18Rα-mediated signaling pathway exacerbates neutrophil infiltration in imiquimod-induced psoriasis murine model

**DOI:** 10.3389/fmed.2023.1293132

**Published:** 2023-10-27

**Authors:** Hiroki Akazawa, Yuji Nozaki, Hirotaka Yamazawa, Kaori Ishimura, Chisato Ashida, Akinori Okada, Koji Kinoshita, Itaru Matsumura

**Affiliations:** Department of Hematology and Rheumatology, Kindai University Faculty of Medicine, Osakasayama, Japan

**Keywords:** immune-mediated inflammatory disease, psoriasis, IL-18Rα, neutrophil, innate immunity

## Abstract

Psoriasis is an immune-mediated inflammatory disease of the skin, which is characterized by epidermal hyperkeratosis and neutrophil infiltration. The interleukin (IL)-17/IL-23 pathway and associated cytokines play major roles in the pathogenesis and exacerbation of psoriasis. The IL-18/IL-18 receptor (R) α signaling pathway is important for Th1 cytokine production and differentiation of Th1 cells; however, its role in the pathogenesis of psoriasis remains unknown. In this study, we investigated the effect of the IL-18Rα-mediated signaling pathway in the pathogenesis of psoriasis in *Il18ra-*deficient mice (*Il18ra*^**−**/**−**^) and wild-type imiquimod (IMQ)-induced psoriatic dermatitis model mice. Blocking this pathway exacerbated IMQ-induced psoriatic skin inflammation. *Il18ra* deficiency led to significant increases in the levels of IL-1β, IL-6, IL-8, IL-17A, IL-23, and chemokine (C-X-C motif) ligand 2 in skin lesions. Gr1-positive cells highly infiltrated psoriatic skin lesions in *Il18ra*^**−**/**−**^ mice compared to those in wild-type mice. Citrullinated histone H3-positive area was relatively broad in *Il18ra*^**−**/**−**^ mice. These results suggest that IL-18Rα-mediated signaling pathways may inhibit psoriatic skin inflammation by regulating infiltration and activation of neutrophil and other innate immune cells.

## 1. Introduction

Psoriasis is an immune-mediated, chronic skin disease that is characterized by excessive epidermal proliferation and intraepidermal infiltration of neutrophils (1). Its prevalence is estimated to be 2%–4%, with males being twice as likely to be affected as women (2, 3). Erythema desquamation affects all skin lesions. Psoriasis significantly impairs patients’ quality of life, treatment satisfaction, adherence, and socioeconomic stability (4). In addition to skin lesions, approximately 30% patients with psoriasis develop psoriatic arthritis, with genetic factors playing a major role in the development of psoriasis (5, 6).

Psoriasis results from the interaction between overgrown keratinocytes and activated infiltrating immune cells (7). The immune system plays an important role in the pathogenesis of psoriasis, as evidenced by the effectiveness of immunosuppressive drugs in ameliorating this disease (8). T cells, specifically helper T cell 1 (Th1) and interleukin (IL)-17-producing helper T cell (Th17), are abundant in psoriatic skin lesions (8, 9). Interferon gamma (IFN-γ)-producing T cells are also increased in psoriasis (10). Moreover, the discovery of Th17 subsets and IL-23 has revealed the involvement of the IL-23/IL-17A axis in the pathogenesis of psoriasis (11, 12). Biological agents targeting IL-23 and IL-17A have shown excellent efficacy in treating psoriasis, implying that the IL-23/IL-17A axis is a major pathway involved in the pathogenesis of psoriasis (13, 14).

IL-18 increases the levels of inflammatory cytokines and chemokines, so called proinflammatory cytokine. This cytokine was originally identified as an inducer of IFN-γ production (15). IL-18 is present intracellularly as a precursor and is released extracellularly in its mature form by cleaving with caspase-1 at the nucleotide-binding domain and by leucine-rich repeat pyrin containing protein (NLRP)-3 inflammasome and other inflammasomes (16). Together with IL-12, IL-18 induces T cells to produce IFN-γ and plays an important role in Th1 response (17, 18). IL-18 receptor alpha chain (IL-18Rα) and IL-18 receptor β chain (IL-1RAcPL, also called IL-1R7) form a combined signaling complex. Following this heterodimer, the Toll/IL-1 receptor domain activates myeloid differentiation primary response 88 (MyD88), IL-1R-associated kinase, and nuclear factor kappa-chain-enhancer of activated B cells (NF-κB) (19). Under Th1 conditions, IL-18 recruit dendritic cells to the inflammatory foci of psoriasis. IL-18 is produced by immune cells, such as Th1 cells, NK cells, macrophages, and keratinocytes, together with IL-12, and plays an important role in psoriatic skin lesions by inducing IFN-γ production (20). In an imiquimod (IMQ)-induced mouse model of psoriasis, IL-18 stimulates Th17 and γδT cells to secrete IL-17 (21). This cytokine may also induce Th1 responses synergistically with IL-23 without inhibiting the IL-23/IL-17 signaling axis or exacerbating inflammation in murine dermatitis (22). However, the mechanism by which the downstream pathway of IL-18Rα contributes to the pathogenesis of psoriasis remains largely unknown.

We herein induced psoriasis in *Il18ra-*deficient (*Il18rα*^−/−^) and wild-type (WT) mice using IMQ to investigate the role of the IL-18/IL-18Rα signaling pathway in pathogenesis and severity of psoriasis.

## 2. Methods

### 2.1. Ethics statement

The animal protocols were approved by the Kindai University Animal Care Committee and performed in accordance with the Kindai University Animal Care Guidelines (KAME-2021-049/2021).

### 2.2. Animals

*Il18ra*-deficient (*Il18ra*^−/−^) C57BL/6 mice were kindly provided by Dr. Shizuo Akira (Osaka University, Osaka, Japan). C57BL/6 mice, used as WT controls, were purchased from the Shizuoka Laboratory Animal Centre (Shizuoka, Japan). All mice were maintained in our specific pathogen-free animal facility.

### 2.3. Murine Model of Imiquimod (IMQ) induced psoriasis

**Figure 1:**
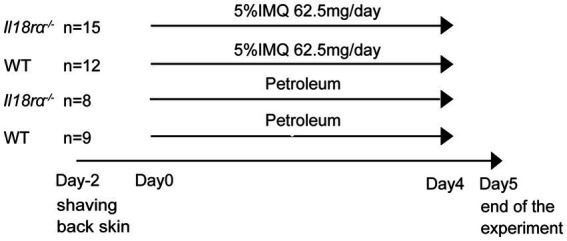
Experiment schedule in wild-type and *Il18ra*^−/−^ mice with psoriasis induced by IMQ. The IMQ cream (5%) or petroleum was applied to the shaved back skin of female *Il18Ra*^−/−^ and WT C57BL/6 mice aged 8–11 weeks consecutively for 5 days (day 0–4). The number of *Il18ra*^−/−^ applied IMQ and petroleum, and WT applied IMQ and petroleum were 15, 8, 12 and 9. *Il18ra*, interleukin-18 receptor alpha; WT, wild type; IMQ, imiquimod.

The back skin of 8–11 weeks-old female *Il18rα*^−/−^ and WT mice were depilated with clippers. After 2 days, mice were treated with IMQ for inducing psoriasis (day 0). From days 0 to 4, 62.5 mg of 5% IMQ cream (Beselna Cream; Mochida, Tokyo, Japan) was applied to the back skin of *Il18rα*^−/−^ and WT mice once daily, and Petroleum was applied to the back skin of *Il18ra*^−/−^ and WT mice as the untreated control (Figure 1). The severity of psoriasis in mice was assessed once a day, as previously described (23). Briefly, physical appearance of mice, including back redness, presence of scales, and skin thickness, was scored in the range of 0–4 using a semi-quantitative scoring system, in which 0, 1, 2, 3, and 4 indicated no, slight, moderate, marked, and very marked, respectively. The cumulative scores of these independent variables were defined as the Adapted-PASI score for mice (24). Mice were sacrificed by carbon dioxide asphyxiation on day 5. Blood was collected in heparinized tubules for measurement of serum cytokines, and skin tissue samples were harvested and spleens were obtained and weighed on day 5.

### 2.4. Histological examination

Back skin tissues obtained from mice on day 5 were cut into sections, fixed in 4% formaldehyde, embedded in paraffin, immersed in Tissue Tech (Sakura Finetek Japan, Tokyo, Japan), and stored at room temperature, while those snap-frozen in liquid nitrogen were stored at −80°C. Paraffin sections (5 μm) were cut for hematoxylin and eosin staining. Skin thickness was measured at three random points. Cryosections (5 μm) were cut using a cryostat (LEICA CM3050 S; Leica Biosystems, Tokyo, Japan) for immunohistochemistry. Sections were fixed in acetone containing 0.3% H_2_O_2_ for 15 min at room temperature. Slides were incubated overnight at 4°C with the following primary antibodies: monoclonal antibody for Gr1/Ly6C (1:100) (Pharmingen, San Diego, CA, United States), rat monoclonal antibody GK1.5 for CD4 (1:100) (Pharmingen), F4/80 hybridoma culture supernatant (1:2.5) (HB 198; American Type Culture Collection, Manassas, MD, United States), mouse monoclonal antibody for CD11c (1:100) (Abcam, Cambridge, United Kingdom), and rabbit polyclonal antibody for citrullinated histone H3 (1:200) (Abcam, Cambridge, United Kingdom). Citrullinated histone H3 was stained to analyze the neutrophil extracellular traps (NETs) (25, 26). Slides were then incubated with biotin- or horseradish peroxidase-conjugated secondary antibodies for 40 min. Images were acquired using an All-in-One Fluorescence Microscope BX-710 (Keyence, Osaka, Japan) for measurement of the positive areas.

### 2.5. Measurements of mRNA expression in the skin by real-time PCR

For extracting skin RNA, total skin RNA was isolated with TRIzol reagent (Invitrogen, Carlsbad, CA) according to the manufacturer’s protocol. The final product was air-dried, solubilized in DNase/RNase-free ultrapure water (Invitrogen), and stored at −80°C. The RNA concentrations were measured by 260 nm spectroscopy. RT-PCR was performed using skin tissue samples. FastStart DNA Master SYBR Green I (Applied Biosystems, Foster City, CA, United States) was used to analyze the mRNA expression of *Il22*, *IL23a*, chemokine (C-X-C motif) ligand 2 (*Cxcl2*), *Caspase1*, *Nlrp3*, retinoic acid receptor-related orphan receptor-gamma t (*Rorc*), forkhead box protein 3 (*Foxp3*), and *Rn18s*. TaqMan Gene Expression Assay (Applied Biosystems) was used to quantify the mRNA expression of *Il1β*, *Il4*, *Il6*, *Il17a*, *Il18*, *Ifng*, Toll-like receptor 4 (*Tlr4*), and *Rn18s*. Primer sequences and Taqman Gene Expression assay ID numbers are listed in Tables 1, 2. The relative amount of mRNA was calculated using the comparative Ct (∆∆Ct) method. All specific amplicons were normalized with respect to *Rn18s* expression.

**Table 1:** Primer sequences used for analysis of mRNA expression.

Gene name	Forward primer	Reverse primer
*Rn18s*	GTAACCCGTTGAACCCCATTC	GCCTCACTAAACCATCCAATCG
*Il22*	TTGACACTTGTGCGATCTCTGA	CGGTTGACGATGTATGGCTG
*Il23a*	CCAGCGGGACATATGAATCTACT	CTTGTGGGTCACAACCATCTTC
*Cxcl2*	CGCCCAGACAGAAGTCATAG	TCCTCCTTTCCAGGTCAGTTA
*Nlrp3*	ATCAACAGGCGAGACCTCTG	GTCCTCCTGGCATACCATAGA
*Caspase1*	CTTGGAGACATCCTGTCAGGG	AGTCACAAGACCAGGCATATTCT
*Rorc*	AGATTGCCCTCTACACG	GGCTTGGACCACGATG
*Foxp3*	TCTTGCCAAGCTGGAAGACT	AGCTGATGCATGAAGTGTCG

**Table 2:** Taqman gene expression assay used for analysis of mRNA expression.

Gene name	TaqMan assay ID
*Rn18s*	Cat. No. 4310893E
*Il1β*	Mm00434228_m1
*Il4*	Mm99999154_m1
*Il6*	Mm00446190_m1
*Il17a*	Mm00439618_m1
*Il18*	Mm00434226_m1
*Ifng*	Mm99999071_m1
*Tlr4*	Mm00445273_m1

### 2.6. Serum cytokines quantification by ELISA

Serum levels of IFN-γ, IL-6, and IL-17A were determined using sandwich ELISA for each cytokine, as previously described (27) ELISA kit was used for IL-6 (BD Bioscience) and commercially available antibody pairs were used for IFN-γ (BD Pharmingen) and IL-17A (Invitrogen). Catalog number are listed Table 3.

**Table 3:** Kit and antibody used for analysis of serum cytokine level.

Cytokine name	Catalog number	
IL-6	555220	
IL-17A	14-7175-81	13-7177-81
INF-γ	551216	554410

### 2.7. Statistical analysis

Data are presented as mean ± standard deviation (SD). For comparison among multiple groups, a non-parametric Mann–Whitney *U* test or analysis of variance (ANOVA) was used. Correlations were evaluated using linear regression analysis. Statistical significance was set at *p* < 0.05.

## 3. Results

### 3.1. *Il18ra* deficiency exacerbates IMQ-induced skin inflammation in mice

**Figure 2:**
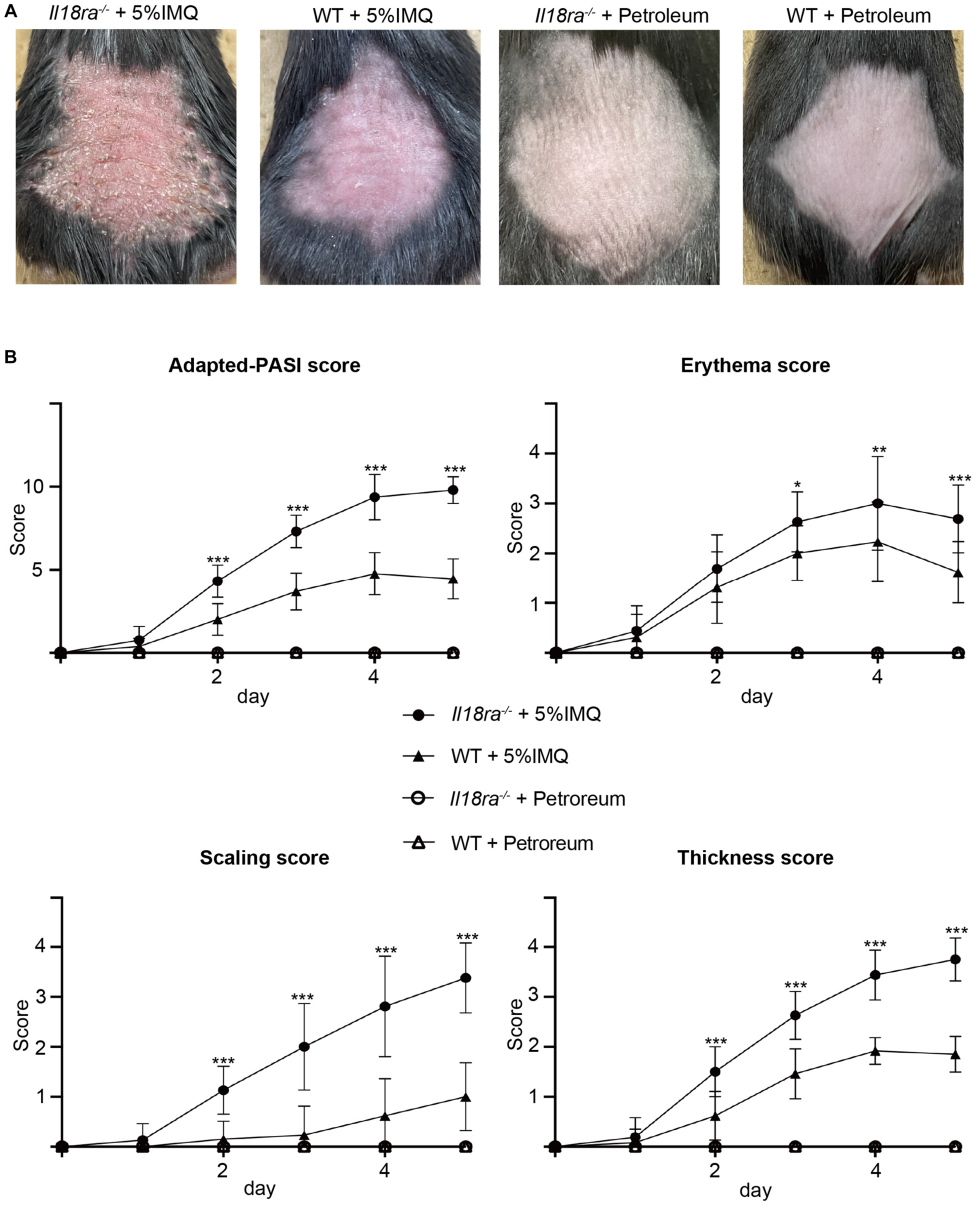
IMQ-induced psoriasis was exacerbated in *Il18ra* deficiency mice were treated with imiquimod cream or petroleum for 5 days **(A)**. Cumulative scores, as PASI score for mice, and each independent score (erythema, thickening, scaling) of *Il18ra*^−/−^ and WT were scored consecutively for 5 days **(B)**. Cutaneous involvement on the back of IL-18Rα^−/−^ or WT mice treated with IMQ or petroleum. Photographs were taken on day 5. Values are mean ± SD. ^*^p < 0.05, ^**^p < 0.01, ^***^p < 0.001. *Il18ra*, interleukin-18 receptor alpha; WT, wild type; IMQ, imiquimod; ns, not significant.

To determine the precise mechanism by which the IL-18Rα-mediated signaling pathway affects psoriasis, we applied IMQ cream on shaved back skin of *Il18ra*^−/−^ and WT mice for five consecutive days to compare the severity of psoriasis between the two groups. Psoriatic lesions, such as erythema, scaling, and thickening, appeared on the back skin of mice-2–3 days after IMQ application (Figure 2A). The cumulative skin score and independent scores for erythema, scaling, and lesion thickness are shown in Figure 2B. The psoriatic lesions in *Il18ra*^−/−^ mice showed much more severe skin erythema, scaling, and thickness than those in WT mice. The median cumulative skin score and independent scores of the *Il18ra*^−/−^ group were significantly higher than those of the WT group on day 5 (total skin score, *Il18ra*^−/−^ 9.8 ± 0.8 vs. WT 4.7 ± 1.2, *p* < 0.0001; erythema, *Il18ra*^−/−^ 2.7 ± 0.7 vs. WT 1.6 ± 0.6, *p* = 0.0004; scaling, *Il18ra*^−/−^ 3.4 ± 0.7 vs. WT 1.0 ± 0.6, *p* < 0.0001; thickness, *Il18ra*^−/−^ 3.8 ± 0.4 vs. WT 1.9 ± 0.4, *p* < 0.0001). The same experiment performed with male *Il18ra*^−/−^ indicated that psoriasis was exacerbated compared to that in WT mice. Their data indicate that the increased severity of the psoriatic phenotype in the *Il18ra* knockout mice is not sex-specific (Supplementary Figure S1).

### 3.2. Antigen-presenting cells are strongly induced in skin lesions

**Figure 3:**
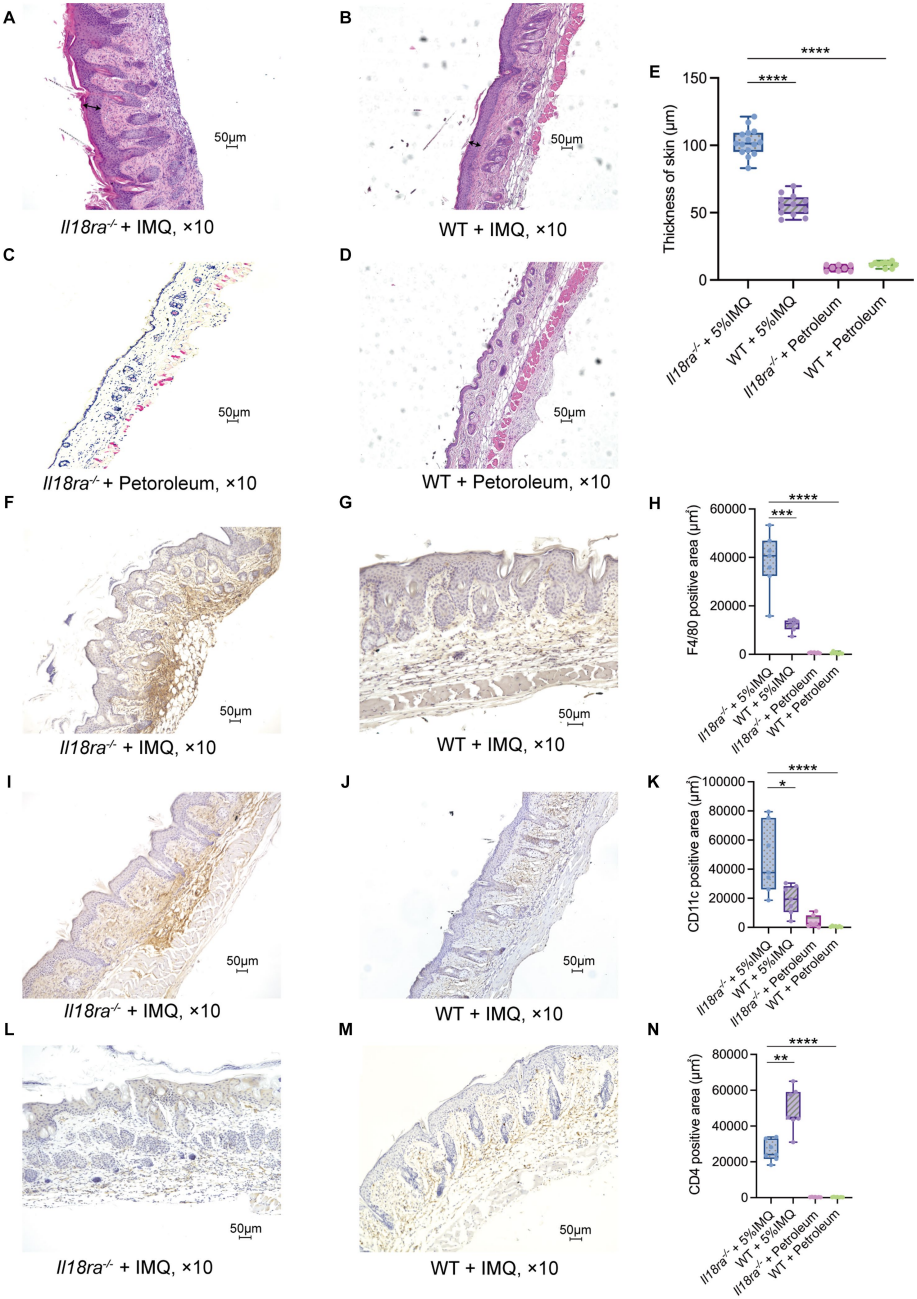
*Il18ra*-mediated signaling pathway suppresses antigen presenting cells (APCs) activities in IMQ-induced psoriasis hematoxylin and eosin (H&E) stained specimens of *Il18ra*^−/−^ (*n* = 15 and 8) and WT (*n* = 12 and 9) skin sections on day 5 after the start of treatment with IMQ or petroleum **(A–D)**. Arrows indicate skin thickness. Skin of IL-18Rα^−/−^ was thicker than WT **(E)**. Increased F4/80 and CD11c positive cell infiltration in *Il18ra*^−/−^ (**F,I**: *n* = 7) than WT (**G,J**: *n* = 7) skin tissues. The *Il18ra*^−/−^ group had significantly higher F4/80 positive **(H)** and CD11c positive **(K)** cells. Conversely, the WT group had significantly higher CD4 positive cells than the *Il18ra*^−/−^ group (**L–N**: *n* = 7). Photomicrographs were taken at 10× magnification. Values are expressed as mean ± SD. ^*^p < 0.05, ^**^p < 0.01, ^***^p < 0.001. *Il18ra*, interleukin-18 receptor alpha; WT, wild type; IMQ, imiquimod.

Hyperplasia of epidermal keratinocytes, elongation of epidermal protrusions, infiltration of inflammatory cells into the dermis and stratum corneum, and other characteristics of psoriasis were more prominent in the *Il18ra*^−/−^ than the WT IMQ-induced psoriasis models (Figures 3A–D). The skin of *Il18ra*^−/−^ mice was thicker than that of the WT group (*Il18ra*^−/−^ 103.2 ± 9.7 μm vs. WT 55.6 ± 6.8 μm, *p* < 0.0001) (Figure 3E). Immunohistochemistry showed more infiltration of F4/80- and CD11c-positive cells in *Il18ra*^−/−^ mice than that of the WT group (F4/80, *Il18ra*^−/−^ 38255.4 ± 11162.8 μm^2^ vs. WT 12021.5 ± 2283.9 μm^2^, *p* = 0.0006; CD11c, *Il18ra*^−/−^ 46803.3 ± 22113.0 μm^2^ vs. WT 19072.1 ± 9074.6 μm^2^, *p* = 0.0175) (Figures 3H,K). This suggests that innate immune cells, such as macrophages and dendritic cells, are involved in the exacerbation of skin lesions in psoriasis of *Il18ra*^−/−^ mice. In contrast, WT mice had relatively high infiltration of CD4-positive cells (*Il18ra*^−/−^ 26072.5 ± 5435.9 μm^2^ vs. WT 49302.1 ± 10844.2 μm^2^, *p* = 0.0023) (Figure 3N). This suggested that acquired immune cells, such as CD4-positive T cells, may be less involved in exacerbating dermatitis in the *Il18ra*^−/−^ model than innate immune cells.

### 3.3. Inhibition of *IL18ra* induces a strong systemic inflammatory response mediated by the innate immune system

**Figure 4:**
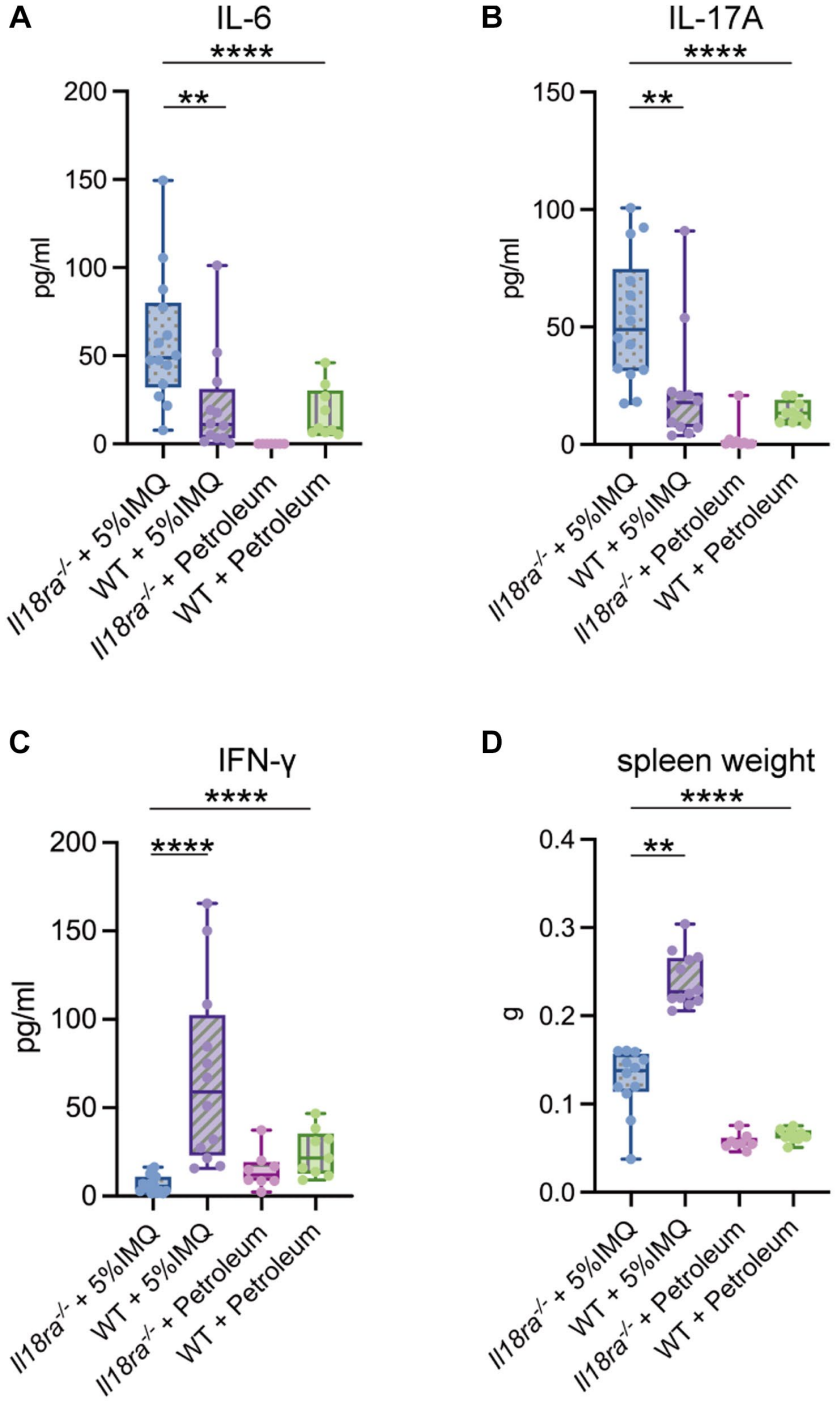
*Il18ra*-mediated signaling pathway suppresses the systemic inflammatory response independent of the Th1 response. Systemic inflammation indicators such as serum IL-17A and IL-6 are highly upregurated in the *Il18ra*^−/−^ group than in the WT group **(A,B)**. Whereas, the adaptive immune response characterized by IFN-γ production is more downregulated than in the WT group **(C,D)**. The data are expressed as mean ± SD. ^*^p < 0.05, ^**^p < 0.01, ^***^p < 0.001. IL-6, interleukin-6; IL-17A, interleukin-17A; *Il18ra*, interleukin-18 receptor alpha; WT, wild type; IFN-γ, interferon gamma; WT, wild type; IMQ, imiquimod.

Serum levels of inflammatory cytokines, such as IL-6, IL-17A, and IFN-γ, were measured using ELISA (Figure 4). IL-6 and IL-17A levels were significantly higher in the *Il18ra*^−/−^ group than in the WT group, inducing a strong systemic inflammatory response (IL-6, *Il18ra*^−/−^ 58.5 ± 35.6 pg/mL vs. WT 21.6 ± 28.1 pg/mL, *p* = 0.0031; IL-17A, *Il18ra*^−/−^ 53.1 ± 26.2 pg/mL vs. WT 23.2 ± 24.1 pg/mL, *p* = 0.0031). In contrast, IFN-γ level was significantly high in the WT group (*Il18ra*^−/−^ 19.0 ± 26.2 pg/mL vs. WT 104.6 ± 108.0 pg./mL, *p* < 0.0001). Inhibition of *Il18ra* did not lead to splenomegaly, an indicator of activation of adaptive immunity (spleen weight, *Il18ra*^−/−^ 0.1 ± 0.02 g vs. WT 0.2 ± 0.04 g, *p* < 0.0001) (Figure 4D). Blockade of *Il18ra* signaling pathway suppressed IFN-γ production and activity of adaptive immune system, but it exacerbates systemic inflammatory responses. These responses are not mainly mediated by the adaptive immune system, e.g., Th1 response.

### 3.4. Upregulation of skin inflammation during *IL18ra* deficiency is mainly mediated by innate immunity

**Figure 5:**
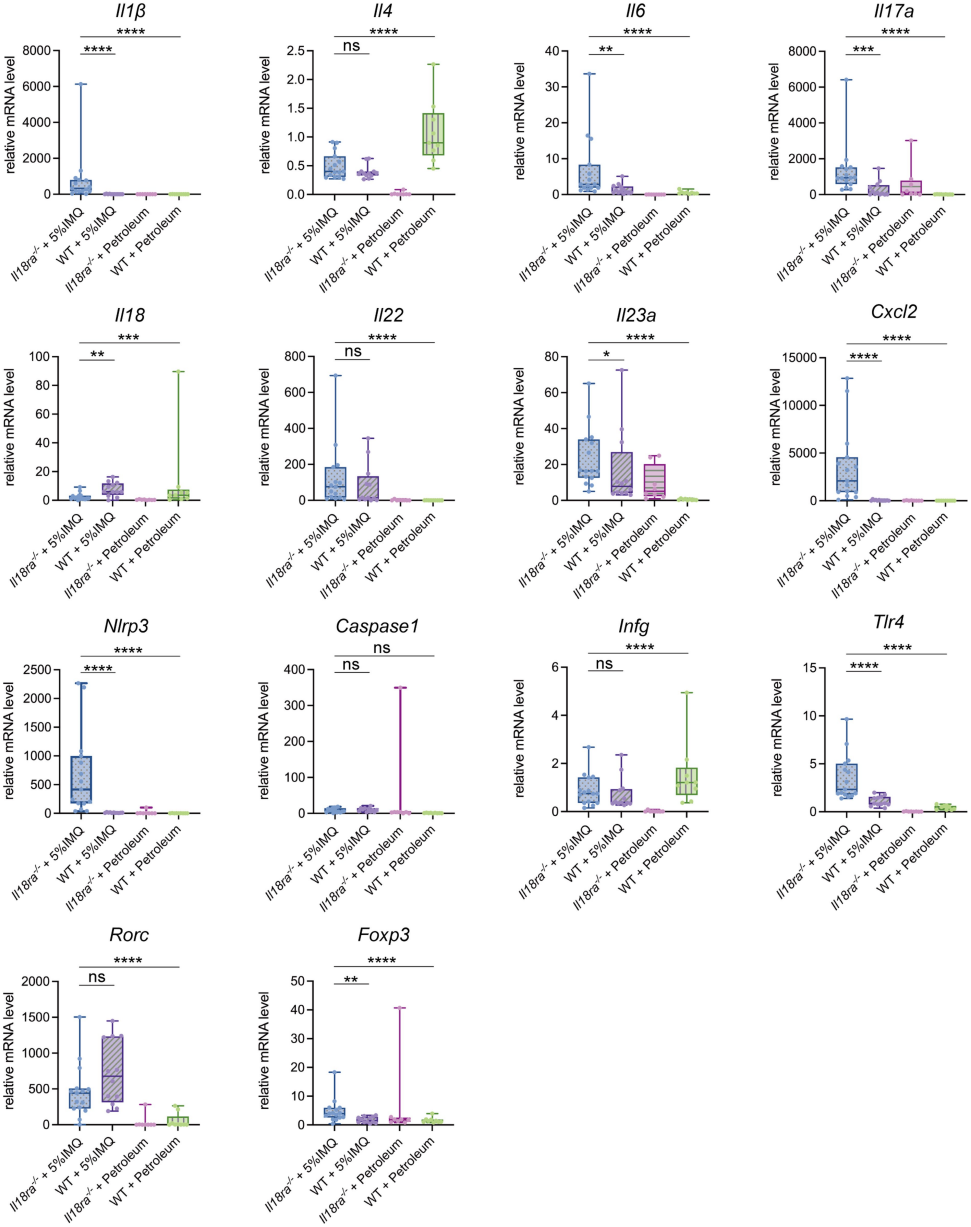
Effects of *Il18ra*-mediated signaling on cytokines and chemokines in dermatitis. Gene expression levels of cytokines, chemokines, transcription factors, Toll-like receptor and inflammasome related proteins in skin lesions were measured by real time PCR. The expression levels were normalized to the expression of *Rn18s* and are expressed relative to the values in untreated control mice. The expression in skin lesions of cytokines involved in the pathogenesis of psoriasis, such as *I17a* and *Il23a*, and chemokines that induce neutrophils to enter lesions, such as *Cxcl2*, was significantly increased in the *IL18ra*^−/−^ group. In addition, there were also differences in the enhancement of cytokines such as *Il1β* and *Nlrp3*, which are involved in the process and outcome of inflammasome activation, suggesting that their activation may be more strongly involved in the exacerbation of dermatitis in the *IL18ra*^−/−^ group than in the WT. The data are expressed as mean ± SD. ^*^p < 0.05, ^**^p < 0.01, ^***^p < 0.001, ^****^p < 0.0001. *Il1β*, interleukin-1 be-ta; *Il4*, interleukin-4; *Il6*, interleukin-6; *Il17a*, interleukin-17a; *Il18*, interleukin-18; *Il18ra*, interleukin-18 receptor alpha; *Il22*, interleukin-22; *Il23a*, interleukin-23a; *Cxcl2*, chemokine (C-X-C motif) ligand 2; *Nlrp3*, nucleotide-binding domain and leucine-rich repeat pyrin containing protein-3; *Ifng*, interferon gamma; *Tlr4*, Toll like receptor 4; *Rorc*, retinoic acid receptor-related orphan receptor-gamma t; *Foxp3*, Forkhead box protein 3; WT, wild type; IMQ, imiquimod.

The mRNA expression of inflammatory cytokines, chemokines, transcription factors, Toll-like receptor, and inflammasomes in psoriatic skin was evaluated by PCR (Figure 5). Inflammatory cytokine, such as IL-6, IL-17 and IL-23p19, expression was significantly higher in skin lesions of the *Il18ra*^−/−^ group than that of the WT group (*Il6*, 6.8 ± 8.6 vs. 1.4 ± 1.4, *p* = 0.0031; *Il17a*, 23.6 ± 19.8 vs. 3.5 ± 2.9, *p* = 0.0001; *IL23a*, 23.6 ± 15.9 vs. 16.8 ± 20.3, *p* = 0.0469). The expression of CXCL2, TLR4, and Foxp3 also increased in the skin of *Il18ra*^−/−^ mice, whereas the level of RORγt increased in the skin of WT mice (*Cxcl2*, 3538.9 ± 3783.3 vs. 39.1 ± 49.4, *p* < 0.0001; *Tlr4,* 3.6 ± 2.3 vs. 1.1 ± 0.5, *p* < 0.0001; *Foxp3*, 4.8 ± 4.1 vs. 1.8 ± 1.0, *p* = 0.0031; *Rorc*, 464.3 ± 364.3 vs. 730.8 ± 433.7, *p* = 0.2174). In inflammasomes, caspase-1 expression was similar between the *IL18ra*^−/−^ and WT groups; however, IL-1β and NLRP3 were upregulated in *IL18ra*^−/−^mice (*Il1β*, 816.5 ± 1465.2 vs. 6.9 ± 9.1, *p* < 0.0001; *Nlrp3*, 667.8 ± 699.0 vs. 10.7 ± 5.3, *p* < 0.0001; *Caspase1*, 8.7 ± 5.6 vs. 9.9 ± 5.2, *p* = 0.54). IL-18 expression was significantly higher in skin lesions of the WT group than that of the *Il18ra*^−/−^ group, and no significant differences in IFN-γ and IL-4 expression was observed between the two groups (*Il18*, 2.5 ± 2.4 vs. 7.5 ± 4.7, *p* = 0.0043; *Ifng*, 0.9 ± 0.6 vs. 0.7 ± 0.6, *p* = 0.4559; *Il4*, 0.5 ± 0.2 vs. 0.4 ± 0.1, *p* = 0.2412). Deficiency of *IL18ra* exacerbated the inflammatory and psoriatic responses in the skin. RT-PCR results suggested that activation of the innate immune system, including neutrophils and macrophage, is involved in this process.

### 3.5. The *Il18ra*-mediated signaling pathway suppresses neutrophil-mediated skin inflammation

**Figure 6:**
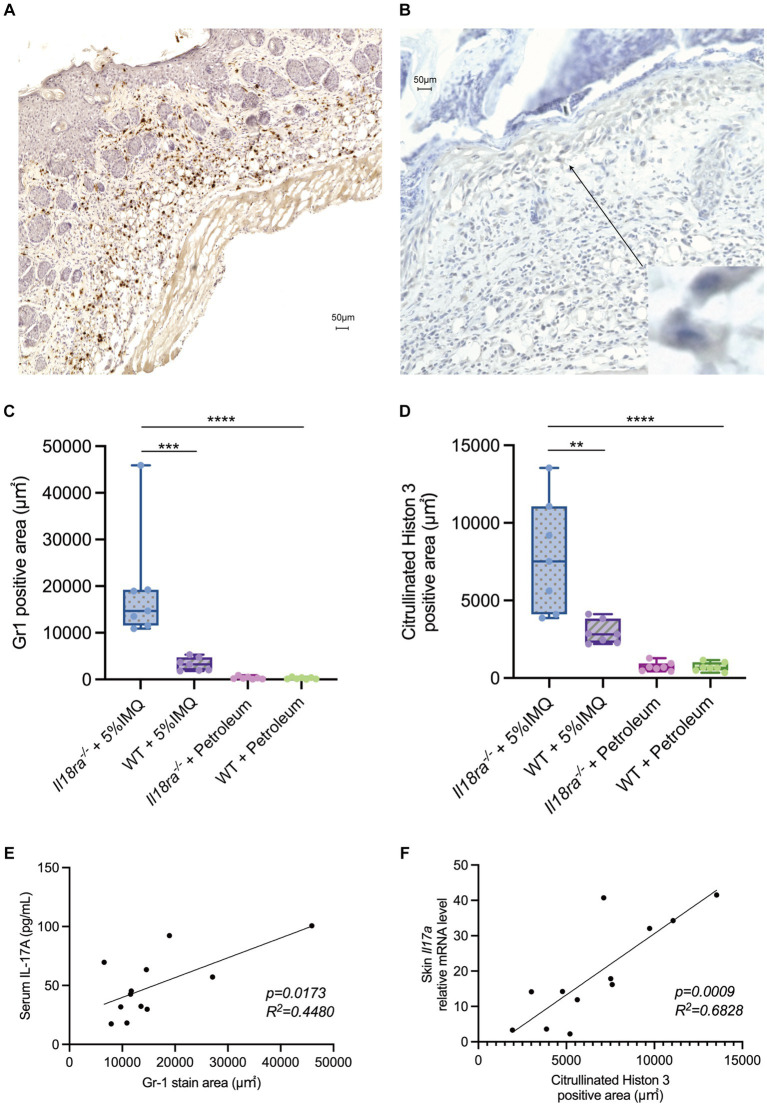
*Il18ra*-mediated signals may suppress IL-17 production by neutrophils activation. Infiltration of Gr1 positive cells and citrullinated histon 3 (NETs) into skin tissues of *Il18ra*^−/−^ applied IMQ **(A,B)**. Photomicrographs were taken at 10× magnification to compare the area of Gr1 positive cells and NETs infiltration into skin lesions between *Il18ra*^−/−^ and WT mice treated with IMQ. Increased infiltration of Gr1 positive cells and NETs in *Il18ra*^−/−^ were observed (**C,D**: *n* = 7). Gr1 positive cell infiltration and serum IL-17A levels correlate moderate and NETs and Il17a mRNA levels in skin lesion also have correlation (Gr1, *R*^2^ = 0.4480, *p* = 0.0173; NETs, *R*^2^ = 0.6828, *p* = 0.0009) (**E,F**: *n* = 12). The data are expressed as mean ± SD. ^*^p < 0.05, ^**^p < 0.01, ^***^p < 0.001, ^****^p < 0.0001. IL-17A, interuikin-17A; l18ra, interleukin-18 receptor alpha; NETs, neutrophil extracellular traps; WT, wild type; IMQ, imiquimod.

Immunohistochemical analysis revealed that inhibition of *Il18ra* results in significantly wider Gr1- and citrullinated histone H3-positive area than that of the WT group (Gr1 positive area, 19247.3 ± 11292.1 μm^2^ vs. 3244.1 ± 1308.1 μm^2^, *p* = 0.0006; citrullinated histone H3 positive area, 7844.4 ± 3369.0 μm^2^ vs. 2931.70 ± 701.7 μm^2^, *p* = 0.0023) (Figures 6A–D). Correlations exist significantly between the Gr1-positive area and serum IL-17 levels and citrullinated histone H3-positive area and *Il17a* mRNA expression in the skin (Figures 6E,F). While the IL-17/23 signaling pathway and neutrophil induction and apoptosis caused by *Il18ra* blockade worsened dermatitis, the expression of *Rorc* was upregulated in the WT group (Figure 5). These results suggest that neutrophils play a major role in the pathway leading to IL-17 production during the pathogenesis and exacerbation of this severe dermatitis. Therefore, in the pathogenesis of IMQ-induced psoriasis, *Il18ra*-mediated signaling may suppress the recruitment and activation of neutrophils to psoriatic lesions and diminish neutrophil-mediated inflammatory responses.

## 4. Discussion

We have previously investigated the involvement of the IL-18Rα-mediated signaling pathways in arthritis and acute kidney injury using *Il18rα* deficient mouse models and found that inhibition of the IL-18Rα-mediated signaling pathway suppresses the production of IFN-γ and IL-6 in models of collagen-induced arthritis and lipopolysaccharide-induced acute kidney injury (27, 28). Additionally, we have observed improvements in the severity of arthritis and survival from acute kidney injury. Similar to previous studies, the inhibition of IL-18Rα suppressed lesional and systemic IFN-γ production in a mouse model of IMQ-induced psoriasis. However, inhibition of the IL-18Rα-mediated signaling pathway increased the production of inflammatory cytokines, such as IL-1β, IL-6, and IL-17, leading to the exacerbation of skin lesions. However, the number of CD4-positive cells and expression of RORγt in skin did not increase by IL-18Rα inhibition. Therefore, the exacerbation of dermatitis by IL-18Rα inhibition was thought to be caused mainly by innate immune cells, such as neutrophil, macrophage and dendritic cells, and not by acquired immune system cells such as Th1 and Th17 cells. Especially neutrophils appeared to have relationship in exacerbating skin lesions.

The IL-23/IL-17 signaling pathway is important for the induction and pathogenesis of psoriasis, as evidenced by the efficacy of IL-17A and IL-23 inhibitors in treating patients with psoriasis (29–31). Although Th17 cells were previously believed to be the major IL-17A-producing cells in psoriatic skin lesions, increasing evidence suggests that neutrophils also play an important role in IL-17A production (32, 33). In particular, patients with severe psoriasis have a higher percentage of activated neutrophils than have healthy individuals or patients treated with biologics, suggesting that neutrophil activation is involved in the pathogenesis of psoriasis, particularly severe psoriasis (34). Treatment with secukinumab, which targets IL-17A, decreases activation and chemokine production in neutrophil-induced skin lesions, indicating that IL-17A from this cell type may be involved in exacerbating psoriasis (35). In addition, the extracellular vesicles of *Staphylococcus epidermidis* ameliorate IMQ-induced psoriasis by reducing the number of Gr1-positive cells infiltrating the skin and IL-17F expression (24). These findings suggest that neutrophil infiltration and neutrophil-derived IL-17 exacerbate psoriatic dermatitis.

In pathologies, such as autoimmune encephalomyelitis, in which Th17 is involved in the pathogenesis, IL-18 upregulate disease activity by inducing IL-17 production from CD4-positive T cells (21). The loss of *Il18ra* reportedly prevents the development of autoimmune encephalomyelitis, implying that signal through IL-18Rα induces the IL-23/IL-17 pathway to potentiate the pathogenesis of autoimmune encephalomyelitis (36). In IMQ-induced psoriasis reported here, inhibition of IL-18Rα exacerbated skin lesions, suggesting that the signaling pathway induced by IL-18Rα may suppress the innate immune response, mainly neutrophil-mediated response in this dermatitis. While IL-18 signaling exacerbates psoriasis pathology by inducing Th1 responses, IL-18Rα mediated pathway may have a regulatory role in neutrophil activation in psoriasis.

IL-37, an IL-1 family cytokine, have an anti-inflammatory role (37). In humans, IL-37 suppresses innate immune activation signaling pathways such as NF-kB and the p38 MAP kinase (MAPK) pathway via the IL-18Rα and IL-1R8 dimer (38). Although it has been reported that dextran-induced colitis inflammation was suppressed in mice that have transduced human IL-37 (39), IL-37 is specific to humans and has not been found in mice. Our study also suggests that there may be a cytokine in the mouse body that has anti-inflammatory effect and downregulate innate immune activity mediated by *Il18ra*, similar to IL-37 in humans.

Neutrophils abundantly release IL-17 when they form NETs (40). LL-37, an antimicrobial component of NETs, forms complexes with DNA and RNA. Keratinocytes in psoriatic lesions are potentially rich in LL-37 and can induce the production of TNF-α and IL-6 in dendritic cells, further indicating that neutrophils are involved in worsening psoriasis (41–43). Keratinocytes are another type of cells involved in the pathogenesis of psoriatic dermatitis. Keratinocytes produce large amounts of chemokines such as CXCL2, CXCL8, and antimicrobial peptides when stimulated with IL-17A (44, 45). Chemokines produced by keratinocytes induce neutrophils, and IL-17A produced by neutrophils further induces keratinocytes to produce chemokines and antibacterial peptides in large amounts. The crosstalk between neutrophils and keratinocytes may be another mechanism underlying the exacerbation of psoriasis. Considering the expression of IL-18Rα in keratinocytes and its role in exacerbating dermatitis, IL-18Rα induced signaling could play a regulatory role in keratinocyte-mediated immune responses.

Psoriasis is an autoinflammatory and autoimmune disorder. Upregulation of the activation of inflammasome and production of IL-1β have been reported in patients with psoriasis. In psoriatic dermatitis, caspase-1 activity increases in skin lesions (46). Patients with psoriasis present relatively high plasma levels of inflammasome-generated IL-1β and high expression of inflammasome components such as NLRP3, NLRP1, and absent in melanoma 2 (AIM2) (47). In our study, the increased infiltration of macrophages into skin lesions and enhancement of IL-1β and NLRP3 expression suggested that inflammasome activation might be involved in exacerbating skin inflammation. Moreover, in chronic airway diseases, such as asthma and chronic obstructive pulmonary disease, extracellular DNA from NETs are associated with high expression of IL-1 and NLRP3 (48). In this severe dermatitis, a pathogenic relationship between NETs and inflammasomes may be considered, which can aggravate the disease condition. Signaling pathway through IL-18Rα might inhibit activation of inflammasome and NET formation.

The present study indicates that IL-18Rα-mediated signaling may modulate the activation of innate immune cells, such as neutrophils and macrophage in severe psoriasis.

Future investigations *in vitro* and signaling pathways downstream of IL-18Rα, such as the activity of intracellular kinase and nuclear transcription factors like NFκB, will help further our understanding of the mechanisms by this pathway to affect innate immune cells such as those cell types in this pathology.

## 5. Limitation

There are some limitations. First, the mouse IMQ model may not represent the whole features of human psoriasis lesions. Mechanisms underlying the pathogenesis, particularly the involvement of keratinocytes, remain unclear, so additional studies are required on the activation of autoinflammation and autoimmunity through the interactions of innate immune cells. Secondary, how γδ T cells, which are known to have predominant roles in psoriasis, work in *Il18ra*-knockout mice was not investigated. Thirdly, the relationship between IL-18Rα and development of psoriatic arthritis, an important complication of psoriasis, has not been researched owing to the short experimental period.

## 6. Conclusion

The IL-18Rα mediated signaling pathway may suppress the activation of innate immune cells, particularly neutrophils and macrophages, in the pathogenesis of IMQ induced psoriatic dermatitis.

## Data availability statement

The original contributions presented in the study are included in the article/Supplementary material, further inquiries can be directed to the corresponding authors.

## Ethics statement

The animal study was approved by Kindai University Animal Care Committee. The study was conducted in accordance with the local legislation and institutional requirements.

## Author contributions

HA: Writing – original draft. YN: Writing – original draft. HY: Writing – original draft. KI: Writing – original draft. CA: Writing – original draft. AO: Writing – original draft. KK: Writing – review & editing. IM: Writing – review & editing.

## Funding

The author(s) declare that no financial support was received for the research, authorship, and/or publication of this article.
