# Supplementary material for: Blockade of IL-18Rα-mediated signaling pathway exacerbates neutrophil infiltration in imiquimod-induced psoriasis murine model
Source: Front Med (Lausanne). 2023 Oct 27;10:1293132. doi: 10.3389/fmed.2023.1293132 (PMC10641785; doi:10.3389/fmed.2023.1293132)
Supplement: Supplementary file 1 [file Data_Sheet_1.docx]

Supplementary Material


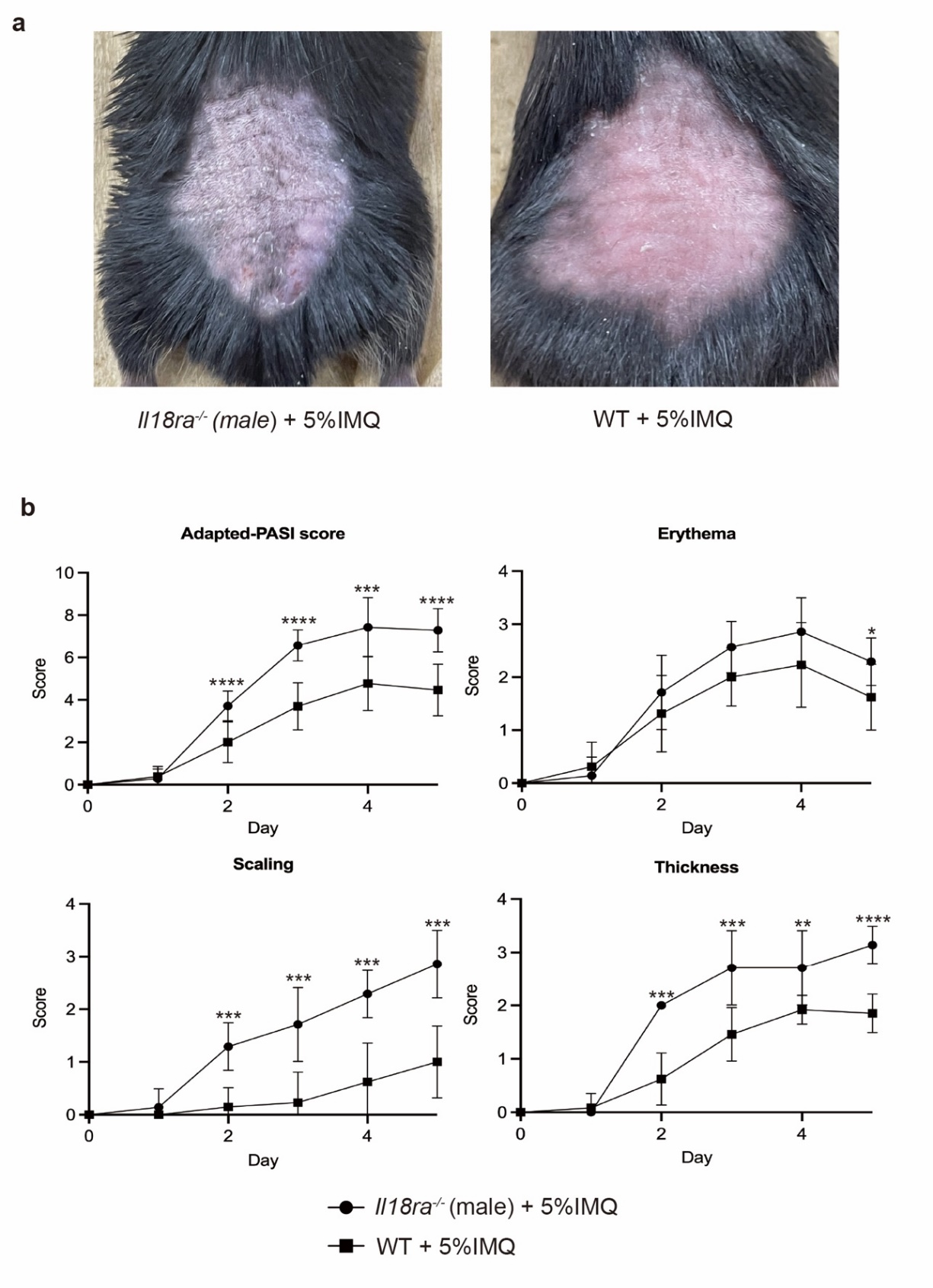


**Supplementary Figure 1.** Male mice of *Il18ra^-/-^* induced severe psoriasis than wild type (WT). *Il18ra*, interleukin-18 receptor alpha; WT, wild type; IMQ, imiquimod.
